# Coronary-to-pulmonary artery fistula in adults: Evaluation with thallium-201 myocardial perfusion SPECT

**DOI:** 10.1371/journal.pone.0189269

**Published:** 2017-12-07

**Authors:** Seul Ki Lee, Jung Im Jung, Joo Hyun O, Hwan Wook Kim, Ho Joong Youn

**Affiliations:** 1 Department of Radiology, Seoul St. Mary’s Hospital, College of Medicine, The Catholic University of Korea, Seoul, Republic of Korea; 2 Department of Nuclear Medicine, Seoul St. Mary’s Hospital, College of Medicine, The Catholic University of Korea, Seoul, Republic of Korea; 3 Department of Thoracic and Cardiovascular Surgery, Seoul St. Mary’s Hospital, College of Medicine, The Catholic University of Korea, Seoul, Republic of Korea; 4 Department of Internal Medicine, Seoul St. Mary’s Hospital, College of Medicine, The Catholic University of Korea, Seoul, Republic of Korea; Ziekenhuisgroep Twente, NETHERLANDS

## Abstract

**Objectives:**

With the increasing use of multi-detector CT, the number of detected cases with coronary-to-pulmonary artery fistula (CPAF) has increased. Several previous studies reported severe cases of angina, but no appropriate tests to evaluate myocardial perfusion for patients with CPAF have been established. We evaluated the hemodynamic characteristics of CPAF using thallium-201 (Tl-201) single photon emission computed tomography (SPECT).

**Materials and methods:**

Tl-201 SPECT was performed in 17 patients with CPAF, but without evidence of coronary artery disease on coronary computed tomography angiography (CCTA) (age, 58.5±13.3 years; 8 men). Quantitative analysis of scintigraphic data was performed. Additionally, perfusion abnormalities were compared with CCTA findings. Medical records were obtained to define clinical data, diagnostic findings, symptoms, management, follow-up data, and major adverse cardiac events (MACE).

**Results:**

Six patients (35.2%) showed perfusion abnormalities on SPECT studies and could be classified as follows: 3 patients, no reversible ischemia (3/17, 17.6%); 1 patient, mild ischemia (1/17, 5.8%); and 2 patients, moderate ischemia (2/17, 11.7%). During the follow-up, ten patients (58.8%) improved under medical management and 5 patients (29.4%) underwent surgical ligation for CPAF with symptomatic improvement in 4 patients. Seven patients performed follow-up myocardial perfusion SPECT, and symptomatic improvement correlated well with scintigraphic perfusion improvement in 6 patients No MACE was observed.

**Clinical significance:**

Tl-201 myocardial perfusion SPECT might be useful for determining the hemodynamic status and for risk stratification in patients with CPAF.

## Introduction

Coronary artery fistula (CAF) is a rare coronary anomaly consisting of an abnormal communication between a coronary artery and a cardiac chamber or the great vessels. With the increasing use of multi-detector computed tomography (MDCT), the number of incidentally found CAF has also increased with incidence upto 0.9% [1, 2]. The most common type of CAF found by MDCT studies is coronary-to-pulmonary artery fistula (CPAF) with prevalence of 0.17–0.67% [1–3]. Several studies reported the prevalence and anatomical features of CPAF detected on MDCT for the purpose of a more accurate diagnosis [1, 2, 4, 5].

However, the clinical implications of incidentally diagnosed CPAF found by MDCT remain unclear. The majority of the adult cases are considered asymptomatic, but some reports showed serious late symptoms or complication such as myocardial ischemia, sudden death, and congestive heart failure [6–15]. The management is controversial and the recommendations of intervention versus medical treatment are based on anecdotal cases or very small retrospective series. A standardized protocol for management of CPAF has not been established yet, owing to its rarity and the variety of specific anatomy and clinical symptoms.

In the absence of established guidelines, it is necessary to discuss the clinical evaluation for patients with CPAF. The natural history of CPAF is not precisely known, but it is likely that small fistulas remain small and moderate fistulas slowly increase in size, although little change might occur over a period of 10 to 15 years. Symptoms tend to develop in the fifth and sixth decades of life [16].

The symptoms or complications of CPAF may be either trivial or lethal. Although the majority of patients with CPAF are asymptomatic or incidentally diagnosed, angina is the most frequent symptom in patients presenting with symptoms [16]. In patients presenting with ischemic symptoms caused by fistula in the absence of coronary artery disease (CAD), the proposed explanation for this discrepancy between the clinical symptoms and anatomical findings is the theory of “coronary steal phenomenon” with coronary blood preferentially passing through the fistula instead of more distal myocardial capillaries [17]. A plausible explanation could be progressive dilatation of the fistula and concomitant increase in the magnitude of the shunt in adult life [16]. Therefore, appropriate tests to evaluate myocardial perfusion are required to prevent late symptoms or life-threatening events.

A nuclear stress test can document ischemia, and may be useful in determining whether myocardial ischemia is associated with CPAF [18]. Although previous studies using myocardial perfusion scintigraphy in patients with CAF reported prevalence of ischemia ranging between 30.4% and 55% [19, 20], few studies reported the prevalence of ischemia with a significant number of patients with CPAF using myocardial perfusion scintigraphy. Only a few cases reported the usefulness of stress/rest myocardial perfusion single photon emission tomography (SPECT) in CPAF [21–24].

Therefore, in our study, we evaluated the hemodynamic characteristics of CPAF using thallium-201 (Tl-201) SPECT. We investigated the prevalence of the perfusion abnormality, objective grade for the severity of myocardial ischemia, and the association between the perfusion abnormality and the morphologic findings determined by coronary computed tomography angiography (CCTA) in patients with CPAF.

## Materials and methods

### Patients

The Seoul St. Mary’s Hospital Institutional Review Board approved this retrospective study and waived the requirement to obtain informed consent. A total of 11,626 patients underwent 12,246 CCTA scans in our institute for various reasons between March 2009 and March 2016. CPAF was found in 72 patients with 104 CCTA scans. The present study included 22 consecutive patients with CPAF who also underwent Tl-201 myocardial perfusion SPECT studies. CCTA was performed within 6 months in all patients with CPAF who performed SPECT studies. All CCTA reports of these patients were reviewed and 5 patients with concomitant CAD were excluded. CPAF was characterized by a communication between 1 or more coronary arteries and the pulmonary artery on the CCTA. CAD was defined as a luminal narrowing of ≥50% found on CCTA.

A total of 17 patients (8 men; mean age, 58.5±13.3 years) with CPAF, without evidence of CAD, and having undergone SPECT studies, were enrolled in this study. Medical records were obtained to define demographic characteristics and history of patients, including age, sex, systolic blood pressure, body mass index (BMI), chief complaints, diabetes mellitus, hypertension, hypercholesterolemia, and cigarette smoking. To further define clinical characteristics of these patients, we attempted to calculate Framingham Risk Score (FRS) to predict 10-year risk of coronary heart disease (angina, myocardial infarction, and coronary death) and patients were categorized into low (<10% in 10 years), intermediate (10% to 20%), and high (>20%) risk categories [25, 26]. Further studies for clarification of clinical presentation or fistula characteristics were performed in the individual patient at the clinician’s discretion. Electrocardiography (ECG) was performed for all patients, and coronary angiography (CAG) was performed for 11 patients.

In order to further investigate the clinical features of these patients, follow-up information was collected, including management, last documented information, major late complication, and the follow-up imaging study. The occurrence of a major late complication was defined as major adverse cardiac events (MACE), which included death, myocardial infarction, and the need for revascularization [27].

### Myocardial perfusion scintigraphy

Myocardial perfusion scintigraphy using Tl-201 was performed in all patients to determine fistula-related ischemia. SPECT study was performed with pharmacologic stress with intravenous adenosine in the dose of 0.56mg/kg over 6 minutes. Approximately 111 MBq of Tl-201 was injected intravenously at 3 minutes. The stress images were acquired 10 minutes after radioisotope injection (Siemens Medical Solutions, Hoffman Estates, IL, USA). Rest images were obtained 3 hours later.

Nuclear medicine physician evaluated SPECT with a semiquantitative segmental scoring system. This visual interpretation of scans reduces the likelihood of overlooking significant defects by differentiating attenuation artifacts. And then quantitative analysis based on the standard 17-segment model for myocardial perfusion SPECT studies were reviewed. The segments were automatically scored by the intensity of radiotracer uptake at each segment using a 5-point scoring system (0, normal; 1, slightly reduced uptake; 2, moderately reduced uptake; 3, severely reduced uptake; and 4, no uptake) by Quantitative Perfusion SPECT (QPS, Cedars-Sinai Medical Center, Los Angeles, CA, USA). Summed stress score (SSS), a quantitative index obtained by the sum of the stress scores of all the segments, and summed rest score (SRS), the sum of the resting scores of all the segments, were computed. Summed difference score (SDS) is the differences between SSS and SRS [28]. The results of SSS were classified as follows: normal for scores <4, mildly abnormal for scores 4–8, moderately abnormal for scores 9–13, and severely abnormal for scores >13 [29]. The SDS indicates the amount of ischemia and the degree of defect reversibility reflecting inducible ischemia. An SDS<2 was considered absence of reversible ischemia, 2–4 was considered mild reversible ischemia, 5–8 moderate reversible ischemia, and >8 severe reversible ischemia [28]. Each of these variables incorporates both the extent and severity of perfusion defects, which independently add prognostic information [30]. We reviewed the SPECT studies for the presence of significant perfusion abnormality, defined as SSS≥4. When present, we assessed the severity of reversible ischemia by SDS.

### Coronary CTA

CCTA scans were performed with a dual-source CT system (Somatom Definition, Siemens Healthcare, Forchheim, Germany). The parameters were as follows: slice collimation 2×32×0.6 mm by means of a z-flying focal spot, gantry rotation time of 330 milliseconds, pitch of 0.2 to 0.5, tube voltage 100–120 kVp (depending on BMI), and the reference tube current of 320 mAs. According to body weight, 60–85 mL of iopromide (Ultravist 370, 370 mg/mL, Bayer Schering Pharma, Berlin, Germany) or Iomeprol (Iomeron 350, 350mg/mL, Bracco, Milan, Italy) at a flow rate of 3.5–5 mL/s, followed by 30–50 mL of contrast mixture (15% contrast medium and 85% saline solution) at the same rate. Contrast material administration was controlled by bolus tracking in the ascending aorta (signal attenuation threshold, 120 HU). The scan delay was 9 seconds. Scans were performed by retrospective ECG-gating method with electrocardiogram-controlled tube current modulation. In the absence of contraindications, patients with a heart rate >80 beats/min received an intravenous selective β1-blocker, esmolol (Brevibloc; Jeil Phama Co., Ltd., Seoul, Korea) before the scan, and a 0.3mg sublingual dose of nitroglycerin was administered just before the scan. Images were reconstructed with a slice thickness of 0.6 mm, a reconstruction increment of 0.5mm, a medium soft-tissue convolution kernel (B26F), and reconstructed matrix size of 512 x512. All images were transferred to a separate workstation equipped with the image processing software (Advantage Window 4.3; GE Healthcare, Milwaukee, WI, USA; Syngo Multimodality Workplace, version 2008; Siemens Healthcare, Erlangen, Germany, Aquarius 3D Workstation, TeraRecon, San Mateo, CA, USA). The effective doses ranged between 3.08 and 14.65mSv (mean dose = 6.62±3.16mSv).

We reviewed the following characteristics of the CPAFs: the number of origin vessel(s), the size (or the diameter) of the largest origin vessel, and the presence of aneurysm. We defined aneurysm as a dilatation 1.5 times larger than the adjacent vessels. After the review of CCTA findings, fistulas were further classified according to their number and size. Singular fistulas between a coronary artery and the pulmonary artery were termed as “single” fistulas, while multiple fistulas between a coronary artery and the pulmonary artery were defined as “multiple” fistulas [19, 31]. We considered that “small” fistulas were little or no dilatation at any point compared to the proximal coronary artery from which they emerged. Fistulas that were similar or larger at any point than the proximal associated coronary artery were considered “large” fistulas [17].

CCTA image analysis for concomitant CAD was performed. In direct accordance with the Society of Cardiovascular Computed Tomography guidelines, coronary segments were visually scored for the presence of coronary plaque by using a 16-segment coronary artery model in an intent-to-diagnose fashion [32]. Only segments with a diameter >1.5 mm were included for analysis. The severity of luminal diameter stenosis was scored as none (0% luminal stenosis), nonobstructive (plaques with maximum stenosis <50%), or obstructive (plaques with maximum stenosis ≥50%). CAD was defined as a luminal narrowing of ≥50% found on CCTA.

### Statistical analysis

All values were expressed as mean±SD. Comparisons between the groups were performed using Student’s *t*-tests for continuous data and Fisher’s exact test for categorical data. *P*-value less than 0.05 was considered statistically significant.

## Results

### Clinical characteristics

The demographic and clinical presentations are summarized in Table 1. The patients’ main clinical presentations were chest pain (n = 13), asymptomatic (n = 2), dizziness (n = 1), and palpitation (n = 1). FRS category was used for the classification of coronary risk stratification: 10 patients were categorized into low risk, 4 patients were classified into intermediate risk, and 3 patients were categorized into high risk according. The ECG revealed atrial fibrillation (AF) in 4 patients and no other abnormalities in 13 patients.

**Table 1:** Clinical data, diagnostic findings, and outcomes in 17 patients with CPAF.

Clinical data						Diagnostic modalities			Outcome	
Case	Sex	Age	Clinical presentation	FRS	ECG	CAG	CCTA	SPECT (SSS/SDS)	Outcome	MACE
1	F	53	chest pain	high	normal	CPAF, no CAD	single, large fistula, no CAD	0/0	controlled by medication	no
2	F	58	chest pain	intermediate	normal	CPAF, no CAD	single, small fistula, no CAD	0/0	controlled by medication	no
3	F	59	asymptomatic	low	normal	N/F	single, small fistula, no CAD	0/0	asymptomatic	no
4	F	64	chest pain	low	AF	CPAF, no CAD	multiple, small fistulas, no CAD	0/0	controlled by medication	no
5	M	63	palpitation	intermediate	AF	CPAF, no CAD	single, large fistula with aneurysm, no CAD	1/1	controlled by medication	no
6	M	67	chest pain	high	normal	CPAF, no CAD	multiple, large fistulas with aneurysm, no CAD	1/1	controlled by medication	no
7	F	77	chest pain	high	AF	CPAF, no CAD	single, small fistula, no CAD	1/1	controlled by medication	no
8	M	41	chest pain	low	normal	CPAF, no CAD	multiple, large fistulas with aneurysm, no CAD	2/0	surgery	no
9	M	49	chest pain	intermediate	normal	N/F	single, small fistula, no CAD	2/2	controlled by medication	no
10	M	60	chest pain	intermediate	normal	N/F	multiple, small fistulas with aneurysm, no CAD	2/2	controlled by medication	no
11	M	40	asymptomatic	low	normal	N/F	multiple, small fistulas with aneurysm, no CAD	3/2	asymptomatic	no
12	F	54	chest pain	low	normal	CPAF, no CAD	multiple, large fistulas with aneurysm, no CAD	4/0	surgery	no
13	M	47	chest pain	low	normal	N/F	multiple, small fistulas, no CAD	4/3	controlled by medication	no
14	F	60	chest pain	low	AF	CPAF, no CAD	multiple, small fistulas, no CAD	4/0	controlled by medication	no
15	F	51	chest pain	low	normal	CPAF, no CAD	multiple, large fistulas with aneurysm, no CAD	6/6	surgery	no
16	F	61	chest pain	low	normal	CPAF, no CAD	multiple, large fistulas with aneurysm, no CAD	6/1	surgery	no
17	M	20	dizziness	low	normal	N/F	multiple, small fistulas with aneurysm, no CAD	7/6	surgery	no

AF = atrial fibrillation; CAD = coronary artery disease; CAG = coronary angiography; CCTA = coronary computed tomography angiography; CPAF = coronary-to-pulmonary artery fistula; ECG = electrocardiography; FRS = Framingham risk score; MACE = major adverse cardiac events; N/F = not performed; SDS = summed difference score; SPECT = single photon emission tomography; SSS = summed stress score.

### Myocardial perfusion scintigraphy

**Fig 1:**
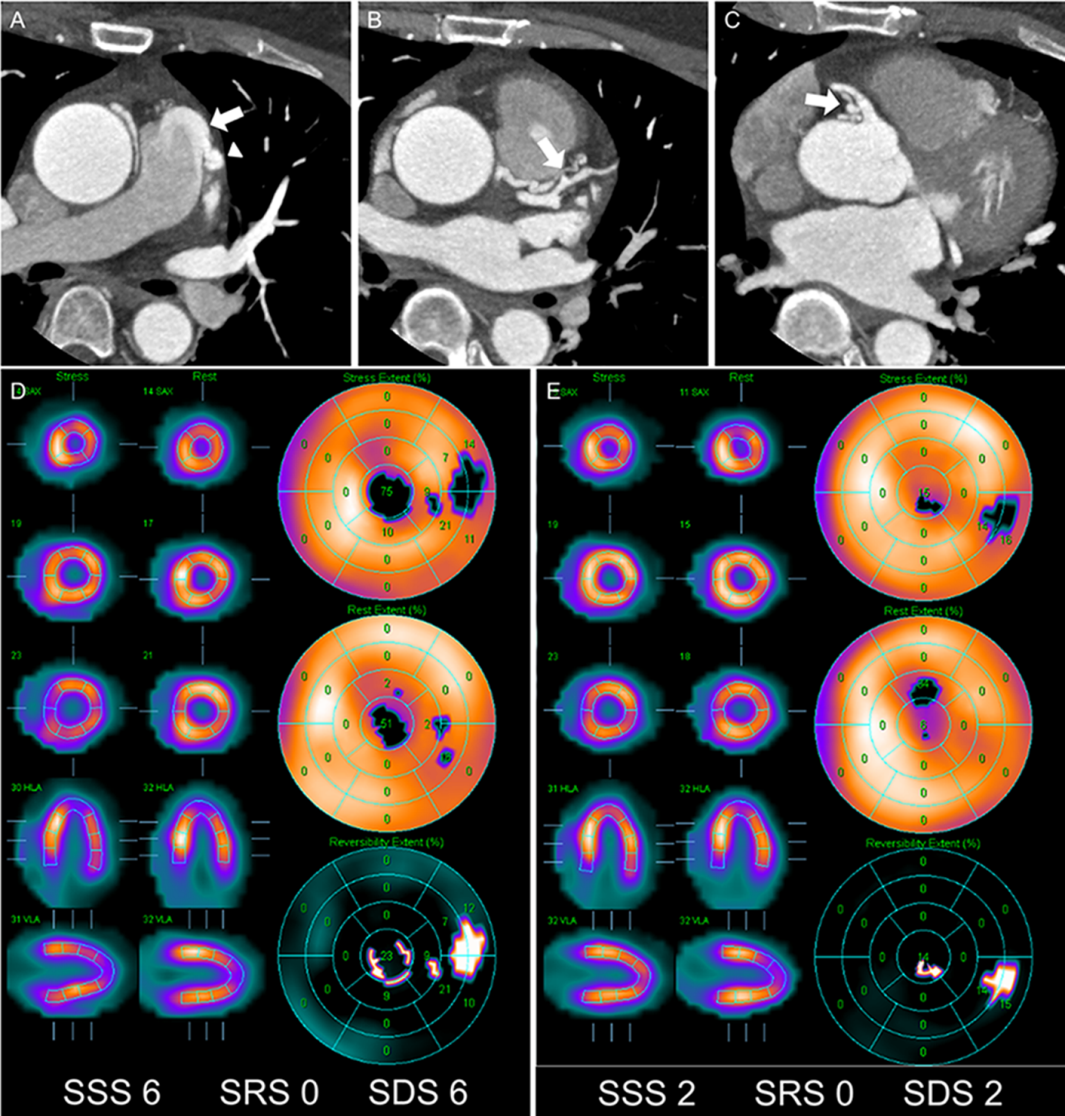
A 52-year-old woman (patient #15) with CPAF who underwent surgical ligation. (A) Axial CCTA images show tortuous and dilated vessels around the main pulmonary artery and a high-density jet flow, which directly inserts into the main pulmonary artery (*arrow*). (B and C) These vessels originate from two different vessels: from the proximal left anterior descending artery (*arrow* in B) and from the proximal right coronary artery (*arrow* in C). (A) This vascular connection passes from the left side of the main pulmonary artery and forms an aneurysmal dilatation (*arrowhead* in A) before it enters the main pulmonary artery. (D) Stress and rest polar maps shows perfusion abnormality (SSS = 6) with moderate reversible ischemia (SDS = 6). (E) After surgical ligation, subsequent SPECT shows decreased extent of the perfusion abnormality (SSS = 2).

**Fig 2:**
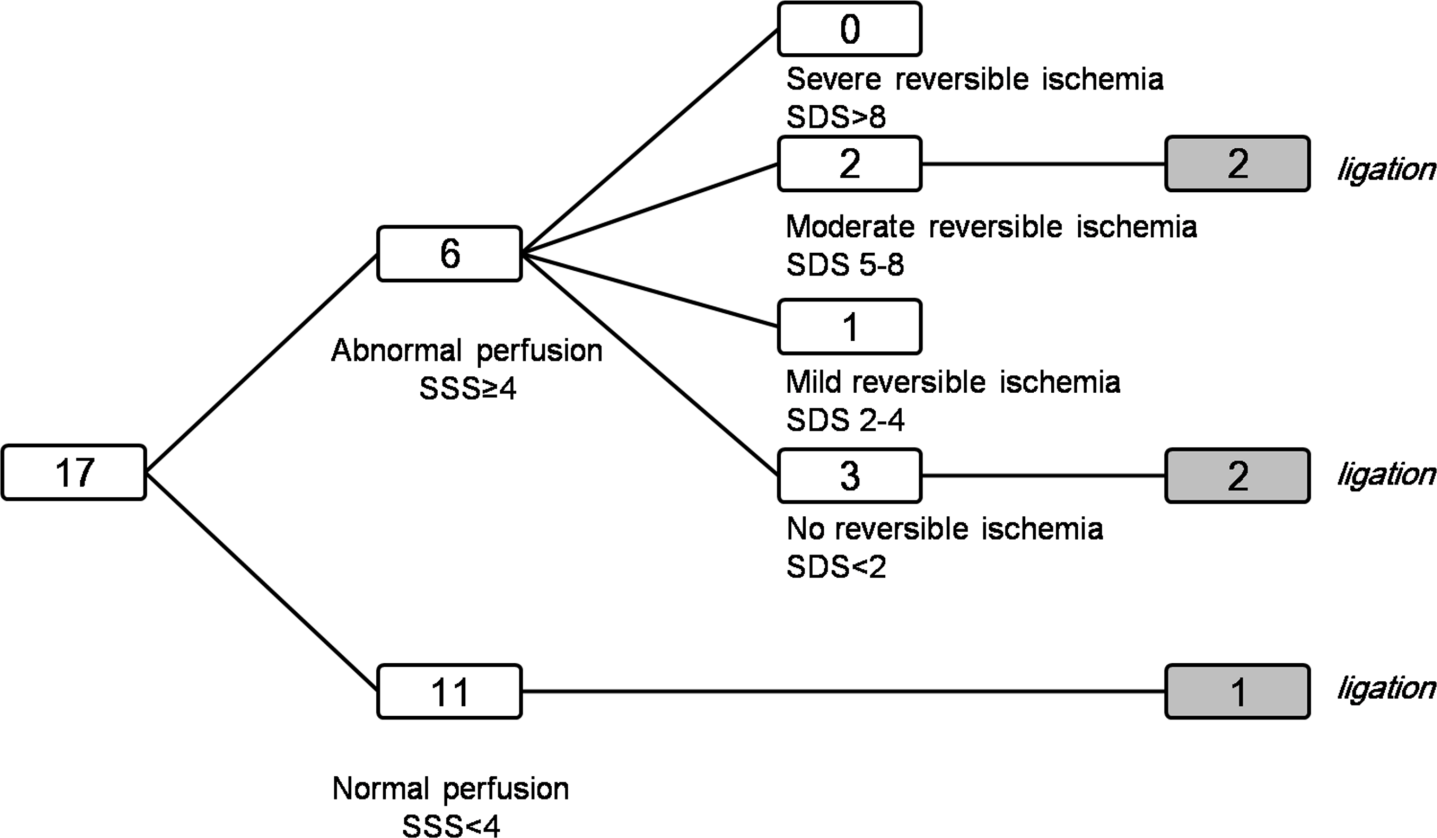
Flow diagram showing the incidence and severity stratification of perfusion abnormalities in 17 patients with CPAF.

The prevalence of perfusion abnormality by QPS with the SSS of 4 or greater was found in 6 patients (35.2%), while the remaining 11 patients (64.7%) showed normal perfusion pattern. Among the 6 patients with abnormal SPECT findings, three patients (50%) had perfusion abnormalities with no reversible ischemia of the SDS less than 2, one patient (16.6%) showed perfusion abnormality with mild reversible ischemia (SDS 3), two patients (33.3%) demonstrated perfusion abnormalities with moderate reversible ischemia (SDS 6, Fig 1), and no patient appeared as perfusion abnormality with severe reversible ischemia by SDS severity stratification. Among the patients with preoperative indications for surgical ligation, perfusion abnormality with moderate reversible ischemia was evidence in 2 patients, perfusion abnormality with no reversible ischemia was observed in 2 patients, and no perfusion abnormality was noted in 1 patient. Fig 2 summarizes the incidence and the severity stratification of perfusion abnormalities in 17 patients with CPAF. Patients with perfusion abnormality and those without perfusion abnormality showed no significant difference in demographic and clinical characteristics except for FRS stratification (Table 2). The number of cardiovascular low risk based on FRS was significantly higher in the abnormal perfusion group compared to that in the normal perfusion group (*P* = 0.03).

**Table 2:** Comparison of patients according to SPECT findings.

	Perfusion abnormality on SPECT, SSS≥4 (n = 6)	No perfusion abnormality on SPECT, SSS<4 (n = 11)	*P*-value
Male/Female	2/4	6/5	0.62
Age (years)	52.5±15.5	61.8±11.4	0.17
Presence of symptoms (%)	6 (100)	9 (81.8)	0.51
Low risk by FRS	6 (100)	4 (36.3)	0.03*
ECG abnormality (%)	1 (16.6)	3 (27.2)	1.00

*P*-value from Fisher’s exact test

* asterisks as indicators for statistical significance.

### Comparison of SPECT and CCTA findings

No significant differences were observed in the CCTA findings between patients with and without perfusion abnormality except in terms of multiplicity (Table 3). The number of origin vessels for abnormal SPECT group was significantly higher than that for normal SPECT group (*P* = 0.04). The prevalence of larger fistulas and aneurysms for abnormal perfusion group (50%, 66.6%, respectively) was higher than that for normal perfusion group (36.3%, 45.4%, respectively), although these differences did not achieve statistical significance.

**Table 3:** Comparison of CCTA findings according to SPECT findings.

	Perfusion abnormality on SPECT, SSS≥4 (n = 6)	No perfusion abnormality on SPECT, SSS<4 (n = 11)	*P*-value
Multiplicity (%)	6 (100)	5 (45.4)	0.04*
Large fistula (%)	3 (50)	4 (36.3)	0.64
Presence of aneurysm (%)	4 (66.6)	5 (45.4)	0.62

*P*-value from Fisher’s exact test.

* asterisks as indicators for statistical significance.

### Treatment and follow-up studies

The follow-up was performed for all included patients. The median period of follow-up was 45 months (2–90 months), and no MACE was observed. Two patients were asymptomatic (11.7%) and the expectative strategy (wait and see) was applied. Ten patients (58.8%) who initially had clinical symptoms subsequently improved under medical management for concomitant disorders (hypertension, 1 patient; hypercholesterolemia, 2 patients; diabetes mellitus, 1 patient; atrial fibrillation, 4 patients; gastroesophageal reflux disease, 1 patient; and asthma, 1 patient). Surgical ligation for CPAF was performed in 5 patients (29.4%). After surgical repair, all patients except one showed symptomatic recovery. One patient still complained of symptoms (chest pain) after surgery and was followed-up with expectative strategy.

The follow-up myocardial perfusion scintigraphy studies were performed in 7 patients (Table 4). Among the patients with follow-up SPECT studies, 3 patients (patient 2, 5, 10) were treated medically, and 4 patients (patient 8, 12, 15, 16) underwent surgical ligation. All patients receiving medical treatment showed no perfusion abnormality on both initial and follow-up SPECT studies. Among the patients with surgical ligation, 3 patients (patient 12, 15, 16) showed perfusion abnormality on preoperative SPECT studies and improvement of both symptoms and perfusion status on postoperative SPECT studies. Of these three patients, patient 15 showed evidence of improvement with regard to severity of perfusion abnormality from moderate reversible ischemia to mild reversible ischemia. However, incomplete recovery of chest pain after surgery was observed in patient 8 who had no evidence of perfusion abnormality on both preoperative and postoperative SPECT studies.

**Table 4:** Initial and post-therapeutic change of SSS and SDS by SPECT studies.

Case	Pre-treatment SPECT		Post-treatment SPECT		Treatment
	SSS	SDS	SSS	SDS	
#2	0	0	0	0	medication
#5	1	1	1	1	medication
#8	2	0	1	1	ligation
#10	2	2	3	3	medication
#12	4	0	0	0	ligation
#15	6	6	2	2	ligation
#16	6	1	1	1	ligation

## Discussion/Conclusions

The clear guidelines for the treatment of CPAF have not yet been established. Therefore, an objective tool for measurement of hemodynamic instability in patients with CPAF is needed. A nuclear stress test may be useful in determining whether myocardial ischemia is associated with CPAF [18]. Several previous cases and studies reported the usefulness of nuclear stress test in patients with CAF or CPAF [19–24, 31, 33, 34]. Myocardial perfusion SPECT was performed traditionally in many previous studies to demonstrate the fistula related myocardial ischemia in patients with CAF or CPAF with prevalence between 30 and 55% to have evidence in the treatment decision [19, 20, 31, 34]. Myocardial perfusion positron emission tomography (PET) is advanced technology with better spatial resolution and sensitivity than SPECT [33, 34]. Said et al. demonstrated the great value of PET scanning to assess the coronary steal phenomenon with flow ratio of regional distribution in patient with CPAF [34]. PET might be promising tool to evaluate the myocardial ischemia in patients with CPAF.

Our study analyzed myocardial perfusion SPECT with the computed values of SSS, SRS, and SDS, which are known to be important both for diagnosis and prognosis [35–37]. We found that prevalence of significant perfusion abnormality in patients with CPAF on SPECT was 35.2% (6/17), which is higher than anticipated. By segmenting, 66.6% (4/6) revealed no or mild reversible myocardial ischemia, which means that most of perfusion abnormality in CPAF is trivial and clinically insignificant.

The prevalence of clinically significant perfusion abnormality (moderate to severe reversible ischemia by SDS stratification) in CPAF was approximately 11.7% (2 out of 17) in our study. The presence of moderate myocardial ischemia in our study raises questions concerning possibility of concurrent cardiovascular risks. However, our results showed that the number of low risk by FRS in abnormal perfusion group was significantly higher than that in normal perfusion group (*P* = 0.03) (Table 2). This result supports the fact that significant perfusion abnormality on SPECT may be actually associated with clinical implication of CPAF rather than other cardiovascular risks.

In our study, among the 6 patients with abnormal SPECT findings, three patients (50%) had perfusion abnormalities with the SDS less than 2, which indicated a perfusion abnormality with no reversible ischemia. The possible causes of perfusion abnormality with absence of reversible ischemia included the following: a) perfusion abnormality with true infarction or fibrosis and b) transient ischemia. Among the three patients, two patients showed normal findings after surgical intervention on postoperative SPECT studies, which means that the initial SPECT studies possibly demonstrated viable (or hibernating) myocardium with transient ischemia. The other patient also revealed transient ischemia according to the serial follow-ups. Sequential follow-up studies with SPECT may support more accurate functional classification of the CPAF.

We speculated that myocardial ischemia may be associated with the CCTA morphologic features of CPAF. We found significant differences in the multiplicity (*P* = 0.04). However, no statistically significant difference was found between the perfusion abnormality and the larger fistulas and aneurysms, although a tendency for patients with perfusion abnormalities to have larger fistulous connection and more frequent aneurysms was noted (Table 3). The number of participants in our study is insufficient to demonstrate the important anatomic factors that are attributed to hemodynamic impairment of CPAF.

In the fistula closure group of four patients with chest pain at initial visit, we found that three patients who had perfusion abnormalities preoperatively (SSS≥4), showed improvement of both symptoms and SPECT findings after the surgery. Through these results, we hypothesized that myocardial perfusion abnormality expressed by SSS on SPECT might be associated with coronary steal phenomenon. In contrast, one patient underwent surgical treatment and showed no improvement of clinical symptoms. This patient showed no evidence of perfusion abnormality, preoperatively. Through this result, we thought that patients with angina, but without perfusion abnormality on SPECT might be further evaluated for other causes of symptom prior to the decision of surgical treatment. Consequentially, we cautiously propose that computed summed scores might be incorporated into the therapeutic decision making process for symptomatic patients. However, it is difficult to set on a specific score as a cutoff-value indicating the need for surgery currently due to the small number of surgically corrected cases in this study. Further studies with larger patient numbers are required.

Our study had several limitations. First, the study population was from a single center, and the number is insufficient to generalize the findings to the actual prevalence of myocardial perfusion abnormality among patients with CPAF. Second, this study was retrospective in design, and a myocardial perfusion SPECT study, if considered according to physicians’ preference due to lack of definite guideline for patients with CPAF, may lead to selection bias. Third, our institution did not perform the functional data with gated studies routinely. Fourth, the reason of association between low risk of FRS and abnormal perfusion finding of SPECT studies was difficult to determine. Lastly, post-therapeutic or follow-up SPECT study was available for only seven patients to evaluate the treatment effect (medication: n = 3, ligation: n = 4)

This study is an exploration study to evaluate the myocardial perfusion SPECT findings and stress and rest scores in patients with CPAF. Perfusion abnormality was detected by SPECT in the absence of CAD in 35.2% of patients with CPAF (6 out of 17). Among the patients with impaired perfusion demonstrated by SPECT, two patients (2/6, 33.3%) had moderate to severe ischemia (11.7% in total 17 patients). In the surgical closure group, symptomatic improvement correlated well with scintigraphic perfusion improvement. Therefore, Tl-201 stress/rest myocardial perfusion SPECT and the computed summed scores may be useful tools for initial risk stratification and guiding the therapeutic management in patients with CPAF.

## Supporting information

S1 File Dataset of this study. (XLSX)
